# Synergistic Interactions Between Leaf Traits and Photosynthetic Performance in Young *Pinus tabuliformis* and *Robinia pseudoacacia* Trees Under Drought and Shade

**DOI:** 10.3390/plants14182825

**Published:** 2025-09-10

**Authors:** Xinbing Yang, Chang Liu, Shaoning Li, Xiaotian Xu, Bin Li, Meng Tian, Shaowei Lu, Na Zhao

**Affiliations:** 1 College of Forestry, Hebei Agricultural University, Baoding 071001, China; yangxinbing2001@126.com (X.Y.); l3hang@163.com (C.L.); tianmeng9805@163.com (M.T.); 2 Institute of Forestry and Pomology, Beijing Academy of Agriculture and Forestry Sciences, Beijing 100093, China; lishaoning@126.com (S.L.); arthurpku@163.com (X.X.); libin@baafs.net.cn (B.L.); 3 Beijing Yanshan Forest Ecosystem Research Station, National Forest and Grassland Administration, Beijing 100093, China

**Keywords:** leaf trait, photosynthetic performance, light environment, drought, ecological adaptation

## Abstract

Spring droughts, increasingly coinciding with canopy shade, interactively stress the growth of urban tree species and are poorly understood in Beijing. Three-year-old saplings of *Pinus tabuliformis* and *Robinia pseudoacacia* were subjected to comparative analysis under four drought–shade sequences, with a full-light, well-watered treatment serving as the control. During two periods encompassing the drought to wilting point and subsequent rewatering, we assessed leaf morphology, water status, photosynthetic gas exchange, and chlorophyll fluorescence. Both species exhibited losses in leaf water and carbon assimilation under drought, yet their adaptive strategies substantially differed. *P. tabuliformis* conserved water through the stable leaf anatomy and conservative stomatal control. In particular, *P. tabuliformis* under full-light and drought conditions decreased their specific leaf area (*SLA*) by 23%, as well as showing reductions in stomatal conductance (*G_s_*) and transpiration rate (*T_r_*) along with the drought duration (*p* < 0.01). As the duration of post-drought rewatering increased, the reductions in the net photosynthetic rates (*P_n_*) of *P. tabulaeformis* showed that the shade condition intensified its photosynthetic limitation and slowed recovery after drought. Under low-light drought, *R. pseudoacacia* exhibited a 52% increase in *SLA* and a 77% decline in *G_s_*; the latter was markedly smaller than the reduction observed under full-light drought. After rewatering, *G_s_* displayed an overcompensation response. The rise in specific leaf area and the greater flexibility of stomatal regulation partly offset the adverse effects of drought. Nevertheless, post-drought *P_n_* recovered to only 40%, significantly lower than the 61% recovery under full-light drought. Moreover, the negative correlation between *SLA* and *P_n_* became significantly stronger, indicating that the “after-effects” of shade–drought hindered photosynthetic recovery once the stress was relieved. Drought duration eroded the phenotypic performance in both species, while the light environment during drought and subsequent rehydration determined the time trajectory and completeness of recovery. These results validate a trade-off between shade mitigation and drought legacy, and guide species selection: plant shade-tolerant *R. pseudoacacia* in light-limited urban pockets and reserve sun-dependent *P. tabuliformis* for open, high-light sites to enhance drought resilience of Beijing’s urban forests.

## 1. Introduction

Global climate change and irregular afforestation have made drought and shading two major bottlenecks restricting tree growth [1,2,3,4]. Beijing is located in a semi-arid region, where approximately 80% of annual precipitation is concentrated in July and August, and the phenomenon of “spring drought” between late spring and early summer has become increasingly prominent [5]. Meanwhile, high-density urban greening, as well as shading from buildings and elevated structures, results in insufficient light in understory and street areas. When water deficit and limited light supply occur simultaneously, leaves, as the core organs for plant carbon assimilation and water regulation and with their synergistic relationship between morphological development and physiological functions, will directly determine the survival and recovery potential of trees [6,7].

Drought and shade exert complex and variable effects on plants, and interspecific variation in adaptive mechanisms is pronounced [8]. When the two stressors act simultaneously, plants must balance growth against survival by flexibly modulating physiological traits such as leaf area, leaf thickness, and photosynthetic efficiency to cope with adverse conditions [9]. Regarding the combined influence of drought and shade on tree photosynthetic physiology, four competing hypotheses have emerged: the interaction hypothesis [10], the trade-off hypothesis [11,12], the facilitation hypothesis [13], and the independent-effect hypothesis [14]. Through multiple regulatory pathways, drought and shade jointly modulate the interplay between leaf traits and photosynthetic–fluorescence physiology [15], enabling plants to sustain growth and survival under severe conditions.

In general, drought reduces the relative water content of plants, induces stomatal closure, and even damages mesophyll cells [16,17,18]. Under shaded conditions, insufficient light limits electron transport and carbon assimilation in plants [19]. However, when these two stressors act simultaneously, plants need to make a trade-off between “morphological plasticity” and “photosynthetic system stability”—that is, adjusting traits such as specific leaf area and leaf dry weight to improve light acquisition efficiency, or adopting a conservative strategy to maintain photosystem activity and carbon assimilation capacity to ensure survival. At present, it is still unclear how different water–light combinations reshape this trade-off relationship, and there is a lack of interspecific comparison of trade-off strategies between urban evergreen trees (*Pinus tabuliformis*) and deciduous broad-leaved trees (*Robinia pseudoacacia*).

The coupling of leaf traits and photosynthetic performance is not a static template but a trade-off curve that constantly “slides” with the water–light environment. Prolonged drought duration or increased drought severity will compress the hydraulic safety margin of leaves, and plants tend to reduce transpiration water consumption by decreasing specific leaf area, thickening leaves, or reducing leaf dry weight [16,17]. In contrast, shading induces an increase in specific leaf area and thinning of leaves to enhance light interception in low-light environments [20]. Although shading can provide space for morphological plasticity, the expanded specific leaf area may be difficult to translate into actual carbon gains due to insufficient substrates for photochemical reactions [21,22]. When the two stressors are superimposed, the “water-saving” demand of drought and the “light-capturing” demand of shading are antagonistic. Therefore, it is of great significance to clarify the correlation and changes between leaf traits and photosynthetic physiological indices under “drought-shading” [4,23,24].

This study takes three-year-old *Pinus tabuliformis* and *Robinia pseudoacacia* saplings as the experimental objects. By simulating shading and drought conditions, the leaf traits, photosynthetic parameters, and chlorophyll fluorescence indicators of the two tree species were measured to characterize the changes in leaf growth and photosynthetic performance under drought and shading conditions, and to study the response patterns and adaptation strategies of the two tree species to water and light constraints. This study aims to provide theoretical support for the protection and restoration of forest ecosystems in the Beijing area under regional climate change, and intends to address the following issues: (1) How do drought and shade interactively alter leaf traits and photosynthetic performance? (2) Does shade during drought modify the trade-off between trait plasticity and photosynthetic efficiency? (3) Which species exhibits stronger resilience under combined stress?

## 2. Results

### 2.1. Effects of Drought and Shade on Leaf Traits of P. tabuliformis and R. pseudoacacia

#### 2.1.1. Specific Leaf Area

Under gradual natural drought stages in all groups, light intensity significantly impacted the changes in the specific leaf area (*SLA*) of both tree species (Table 1). In the full-light groups (T_1_, T_2_), the *SLA* in the saplings of *P. tabuliformis* and *R. pseudoacacia* decreased by 23.14% and 15.95%, respectively, compared to the control (CK). In the low-light groups (T_3_, T_4_), the *SLA* of both species increased with drought intensity. However, the increases were significantly greater in *R. pseudoacacia* than in *P. tabuliformis*.

The significant differences in recovery capacity among species were investigated in the rewatering stages in this study (Table 1). In *P. tabuliformis*, *SLA* gradually recovered to the pre-drought level during the post-drought rehydration in the T_3_ and T_4_ groups, which remained lower than the control levels in the T_1_ and T_2_ groups. Compared to non-shaded stages, shading during both the drought and rehydration stages increased *SLA* recovery in *R. pseudoacacia*.

#### 2.1.2. Relative Leaf Water Content

**Figure 1:**
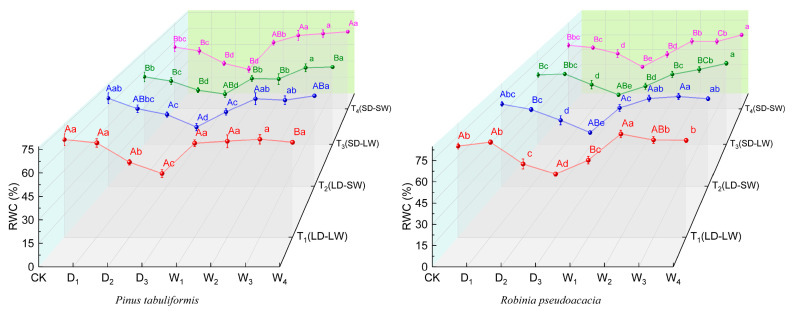
Leaf relative water content (*RWC*, %) of *P. tabuliformis* and *R. pseudoacacia* leaves under four treatment groups during continuous drought and subsequent rewatering. (**left**) *P. tabuliformis*. (**right**) *R. pseudoacacia*. The data is the mean ± standard deviation. D_x_ represents the drought stage, and W_x_ represents the rehydration stage. Different uppercase letters in the figure indicate significant differences among T_1_, T_2_, T_3_, and T_4_ at each sampling node at the *p* < 0.05 level, while different lowercase letters indicate significant differences within the same group at different nodes at the *p* < 0.05 level.

Under the drought stress, both species showed a decrease in leaf relativewater content (*RWC*) (Figure 1). In the full-light drought groups (T_1_, T_2_), *RWC* decreased by 33.23% for *P. tabuliformis* and by 25.1% for *R. pseudoacacia*, while it declined by 30.51% and 31% in the low-light drought groups (T_3_, T_4_), respectively.

The post-drought rehydration highlighted the interspecies differences in *RWC* recovery capacity (Figure 1). In *P. tabuliformis*, *RWC* in the T_1_ group recovered to the control (CK) levels first, followed by T_3_ and T_4_, with T_2_ being the last to recover. In all treatment groups, *RWC* in *R. pseudoacacia* eventually recovered to the control levels, though the recovery timelines varied among the groups. The recovery of *RWC* for *R. pseudoacacia* to control levels took longer in T_3_, T_4_ groups than those in T_1_/T_2_ groups after drought rewatering.

#### 2.1.3. Leaf Dry Weight

**Figure 2:**
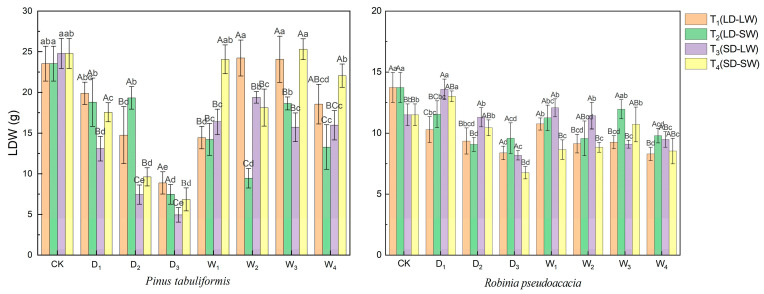
Leaf dry weight (*LDW*, g) of *P. tabuliformis* and *R. pseudoacacia* leaves under four treatment groups during continuous drought and subsequent rewatering. (**left**) *P. tabuliformis*. (**right**) *R. pseudoacacia*. The data is the mean ± standard deviation. D_x_ represents the drought stage, and W_x_ represents the rehydration stage. Different uppercase letters in the figure indicate significant differences among T_1_, T_2_, T_3_, and T_4_ at each sampling node at the *p* < 0.05 level, while different lowercase letters indicate significant differences within the same group at different nodes at the *p* < 0.05 level.

Drought stress significantly suppressed the leaf dry weight (*LDW*) accumulation in both species (Figure 2). In full-light stages, the *LDW* of *P. tabuliformis* decreased by 65.25%, while that of *R. pseudoacacia* decreased by 34.55%. Under shaded–drought conditions, the *LDW* of *P. tabuliformis* further dropped by 76.20%, while that of *R. pseudoacacia* decreased by 34.99%.

The two species exhibited divergent sensitivity to post-drought light environments in LDW recovery dynamics (Figure 2). *LDW* in the consistent light groups (T_1_, T_4_) was 24.92% higher than that in the altered light conditions (T_2_, T_3_) by the end of rehydration. In contrast, *R. pseudoacacia* exhibited superior *LDW* recovery under the low-light rehydration conditions. T_2_, T_4_ groups (low-light rehydration) had 12.61% higher *LDW* than T_1_, T_3_ groups (full-light rehydration) at the endpoint.

### 2.2. Effects of Drought and Shade on Photosynthetic Parameters of P. tabuliformis and R. pseudoacacia

#### 2.2.1. Photosynthetic Rate

**Figure 3:**
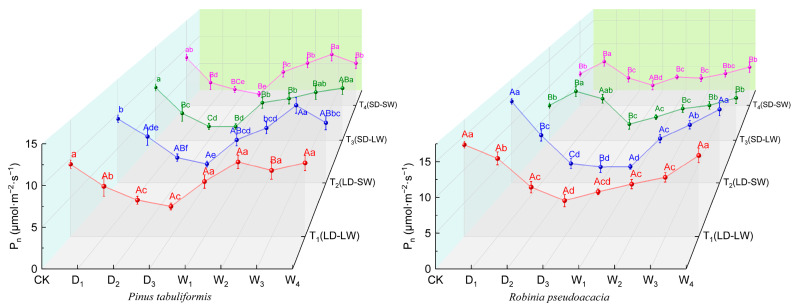
Photosynthetic rates (*P_n_*, µmol·m^−2^·s^−1^) of *P. tabuliformis* and *R. pseudoacacia* leaves under four treatment groups during continuous drought and subsequent rewatering. (**left**) *P. tabuliformis*. (**right**) *R. pseudoacacia*. The data is the mean ± standard deviation. D_x_ represents the drought stage, and W_x_ represents the rehydration stage. Different uppercase letters in the figure indicate significant differences among T_1_, T_2_, T_3_, and T_4_ at each sampling node at the *p* < 0.05 level, while different lowercase letters indicate significant differences within the same group at different nodes at the *p* < 0.05 level.

During drought progression, the two species showed divergent patterns in photosynthetic responses (Figure 3). By the final drought stage, *P. tabuliformis* and *R. pseudoacacia* in the full-light groups (T_1_, T_2_) exhibited reductions of 64.41% and 70.86% in the photosynthetic rate (*P_n_*), respectively. Notably, *P. tabuliformis* in shading drought conditions (T_3_, T_4_) experienced greater declines in *P_n_* of 76.82%, while *R. pseudoacacia* in the low-light groups showed the relatively lower reduction of 44.78%.

The rehydration process in this study revealed the distinct stress-memory effects on the photosynthesis derived from the pre-drought light exposure (Figure 3). In *P*. *tabuliformis*, low-light rewatering groups (T_2_, T_4_) attained the peaks of the *P_n_* during W_3_, whereas the full-light rewatering groups (T_1_, T_3_) only achieved the maximal recovery by W_4_. In contrast, non-shaded drought groups (T_1_, T_2_) of *R. pseudoacaci* exhibited a 61.45% increase in *P_n_* from drought-end to rehydration completion, significantly outperforming the shaded–drought groups (T_3_, T_4_), which only achieved a 39.98% increase.

#### 2.2.2. Stomatal Conductance

**Figure 4:**
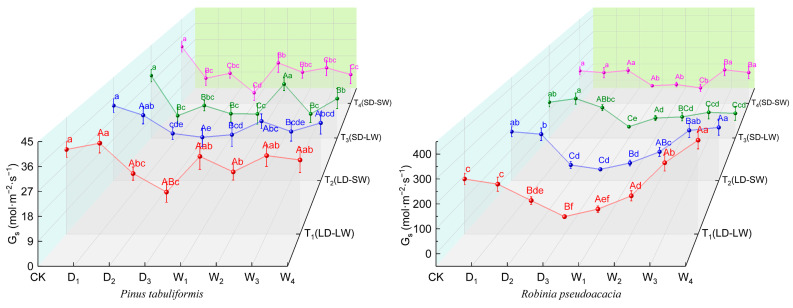
Stomatal conductance (*G_s_*, mol·m^−2^·s^−1^) of *P. tabuliformis* and *R. pseudoacacia* leaves under four treatment groups during continuous drought and subsequent rewatering. (**left**) *P. tabuliformis*. (**right**) *R. pseudoacacia*. The data is the mean ± standard deviation. D_x_ represents the drought stage, and W_x_ represents the rehydration stage. Different uppercase letters in the figure indicate significant differences among T_1_, T_2_, T_3_, and T_4_ at each sampling node at the *p* < 0.05 level, while different lowercase letters indicate significant differences within the same group at different nodes at the *p* < 0.05 level.

During drought progression, stomatal conductance (*G_s_*) responses in both species showed significant interactions with the light environment (Figure 4). Under full-light, *P. tabuliformis* exhibited a 46.33% reduction in *G_s_*, whereas *R. pseudoacacia* exhibited a reduction of 92.16%. In shading drought conditions, declines in *G_s_* intensified to 71.19% in *P. tabuliformis* and 77.29% in *R. pseudoacacia*.

The rehydration after drought highlighted the species-specific adaptive differences in recovery patterns (Figure 4). In *P. tabuliformis*, *G_s_* recovery was significantly lower in low-light drought groups (T_3_, T_4_) compared to full-light drought groups (T_1_, T_2_). In contrast, *R. pseudoacacia* in full-light drought groups (T_1_, T_2_) exceeded the *G_s_* in CK by 50.09%, while shading drought groups (T_3_, T_4_) remained 24.61% below the controls.

#### 2.2.3. Transpiration Rate

**Figure 5:**
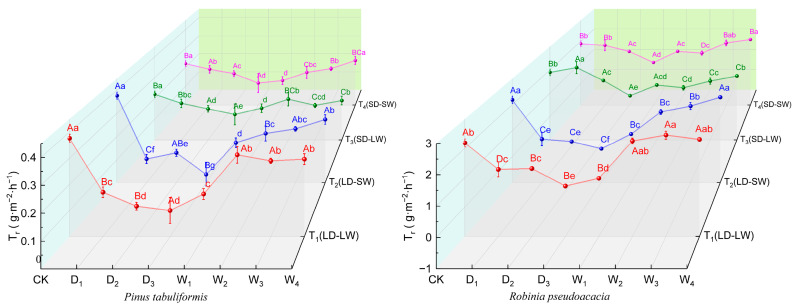
Transpiration (*T_r_*, g·m^−2^·h^−1^) of *P. tabuliformis* and *R. pseudoacacia* leaves under four treatment groups during continuous drought and subsequent rewatering. (**left**) *P. tabuliformis*. (**right**) *R. pseudoacacia*. The data is the mean ± standard deviation. D_x_ represents the drought stage, and W_x_ represents the rehydration stage. Different uppercase letters in the figure indicate significant differences among T_1_, T_2_, T_3_, and T_4_ at each sampling node at the *p* < 0.05 level, while different lowercase letters indicate significant differences within the same group at different nodes at the *p* < 0.05 level.

Under drought stress, both *P. tabuliformis* and *R. pseudoacacia* exhibited significant reductions in transpiration rate (*T_r_*) with the distinct light environment interactions (Figure 5). In full-light conditions, *P. tabuliformis* showed an 81.85% reduction in *T_r_*, which reduced to 77.05% in *R. pseudoacacia*. These reductions were 45.45% in *P. tabuliformis* and 49.82% in *R. pseudoacacia* in the shading–drought conditions.

During rewatering after drought, *P. tabuliformis* in the shaded drought groups (T_3_, T_4_) fully restored the *T_r_* to the control levels, while the full-light drought groups (T_1_/T_2_) remained 27.05% below the controls (Figure 5). Although all treatment groups for *R. pseudoacacia* restored the *T_r_* to the control levels, the consistently low-light rewatering group (T_4_) exhibited 17.22% higher *T_r_* than the light-altered group (T_3_) in the rewatering stages.

### 2.3. Effects of Drought and Shade on Fluorescence Parameters of P. tabuliformis and R. pseudoacacia

**Figure 6:**
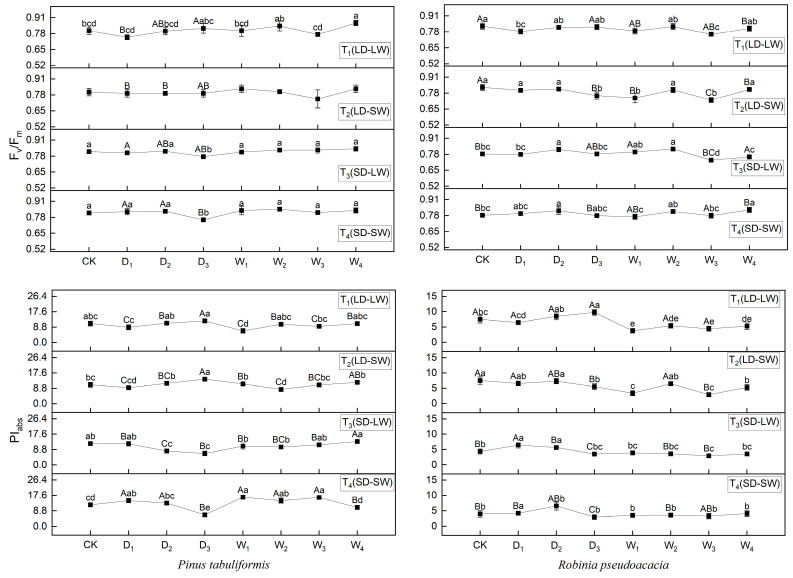
Photochemical efficiency (*F_v_/F_m_*) and photosynthetic performance index (*PI_abs_*) of *P. tabuliformis* and *R. pseudoacacia* leaves under four treatment groups during continuous drought and subsequent rewatering. (**left**) *P. tabuliformis*. (**right**) *R. pseudoacacia*. The data is the mean ± standard deviation. D_x_ represents the drought stage, and W_x_ represents the rehydration stage. Different uppercase letters in the figure indicate significant differences among T_1_, T_2_, T_3_, and T_4_ at each sampling node at the *p* < 0.05 level, while different lowercase letters indicate significant differences within the same group at different nodes at the *p* < 0.05 level.

During drought, shading significantly affected the maximum photochemical efficiency and photosynthetic performance index (*PI_abs_*) of *P. tabuliformis*, with marked differences between full-light (T_1_, T_2_) and shaded (T_3_, T_4_) groups. *PI_abs_* increased under full-light conditions but decreased under shaded conditions. For *R. pseudoacacia*, *PI_abs_* was also significantly influenced by shading, showing substantial differences between full-light (T_1_, T_2_) and shaded (T_3_, T_4_) groups (Figure 6).

During rehydration, both species’ *PI_abs_* were essentially restored to control levels. However, significant differences in *PI_abs_* remained between the previously full-light (T_1_, T_2_). and shaded–drought (T_3_, T_4_) groups for both species by the end of rehydration.

### 2.4. Synergistic Effects of Drought and Shade on Plant Performance—Interaction Analysis

As shown in Table 2, during the drought stage, the *SLA*, *LDW*, *G_s_*, *F_v_/F_m_,* and *PI_abs_* of *P. tabuliformis* were significantly affected by light conditions; during the rewatering stage, light conditions significantly influenced the recovery of *LDW*, *P_n_*, and *PI_abs_*. During the drought stage, the *LDW* and *F_v_/F_m_* of *R. pseudoacacia* were significantly affected by light conditions; during the rewatering stage, these two parameters also showed significant recovery.

A simple effect analysis was conducted on the indicators with significant interactions in Table 2. The results show (Tables S1–S4) that regardless of the light conditions, the key indicators (*SLA*, *LDW*, *G_s_*, *P_n_*, *F_v_/F_m_*, *PI_abs_*) of the two tree species during the drought duration and the rehydration stage mostly show significant differences over time, indicating that the drought stage, rehydration time, and light jointly determine the physiological response trajectory.

### 2.5. Correlations Between Drought-Restoration Duration and Plant Physiological Indicators

#### 2.5.1. Correlations Between Drought-Rehydration Duration and Physiological Indicators in *P. tabuliformis*

The shading stages during drought showed significantly different physiological effects (Table 3). In full-light drought conditions (T_1_, T_2_), dry matter accumulation and photosynthetic capacity were affected, with drought duration (D_n_) showing highly significant negative correlations (*p <* 0.01) with most physiological parameters (*RWC*, *LDW*, *G_s_*, *T_r_*). Under shading (T_3_, T_4_), drought duration not only significantly affected photosynthetic parameters but also showed more pronounced negative correlations with chlorophyll fluorescence parameters (T_3_, D_n_-*F_v_/F_m_*: −0.578 *, D_n_-*PI_abs_*: −0.884 **; T_4_, D_2–3_-*F_v_/F_m_* and *PI_abs_*: −0.974 **).

Shading during drought significantly influenced physiological responses (Table 3). Under full-light drought (T_1_, T_2_), D_n_ showed highly significant negative correlations (*p <* 0.01) with most physiological parameters (*RWC*, *LDW*, *G_s_*, *T_r_*). Under shaded conditions (T_3_, T_4_), drought duration not only significantly affected photosynthetic parameters but also showed more pronounced negative correlations with chlorophyll fluorescence parameters (T_3_, *D_n_*-*F_v_/F_m_*: −0.578 *; D_n_-*PI_abs_*: −0.884 **; T_4_, D_2–3_-*F_v_/F_m_*: −0.974 **, D_2–3_-*PI_abs_*: −0.974 **).

The impacts of light conditions during rehydration on post-drought physiological recovery in *P. tabuliformis* varied significantly (Table 3). Full-light rehydration (T_1_) significantly enhanced short-term transpiration recovery (W_0.25–0.5_-*T_r_*: 0.959 **). By contrast, low-light rehydration promoted superior long-term transpiration recovery (T_2_, W_n_-*T_r_*: 0.658 *). Comparing rehydration stages after shaded drought (T_3_, T_4_), full-light rehydration (T_3_) rapidly activated short-term stomatal recovery (W_0.25–0.5_-*G_s_*: 0.955 **). In T_4_, prolonged low-light rehydration had highly significant negative correlations with *P_n_* and *PI_abs_* (W_1–7_-*P_n_*: −0.923 **, W_1–7_- *PI_abs_*: −0.959 **).

The legacy effects of pre-drought light conditions differentially influenced rehydration outcomes (Table 3). Comparing full-light rehydration groups with pre-drought light exposures (T_1_, T_3_), plants with full-light drought exposure showed better photosystem stability recovery (T_1_, W_1–7_-*F_v_/F_m_*: 0.953 **) than those with shaded drought. Analysis of low-light rehydration groups (T_2_, T_4_) revealed that prolonged low-light rehydration consistently impaired *P_n_* in *P. tabuliformis* (T_2_, W_1–7_-*P_n_*: −0.918 **; T_4_, W_1–7_-*P_n_*: −0.923 **).

#### 2.5.2. Correlations Between Drought-Rehydration Duration and Physiological Indicators in *R. pseudoacacia*

The physiological impacts of shading during drought varied (Table 4). Under full-light drought (T_1_, T_2_), drought duration (D_n_, D_1–2_) showed highly significant negative correlations (*p* < 0.01) with most physiological parameters (*RWC*, *LDW*, *P_n_*, *G_s_*, *T_r_*). Conversely, shaded drought stages promoted dry matter accumulation (T_3_, D_n_-*SLA*: 0.963 **; T_4_, D_n_-*SLA*: 0.958 **). Prolonged shaded drought still exacerbated photosystem damage (T_3_, D_2–3_-*PI_abs_*: −0.904 *; T_4_, D_2–3_-*PI_ab_*: −0.901 *).

The impacts of light conditions during rehydration on post-drought physiological recovery in *R. pseudoacacia* varied significantly (Table 4). Comparing rehydration stages after full-light drought (T_1_, T_2_), prolonged low-light rehydration more effectively restored the light–energy utilization system (T_1_, W_1–7_-*F_v_/F_m_*: 0.868 *; T_2_, W_1–7_-*F_v_/F_m_*: 0.956 **, W_1–7_-*PI_abs_*: 0.870 *). Comparing rehydration stages after shaded drought (T_3_, T_4_), low-light rehydration showed a highly significant positive correlation between W_1–7_ and *P_n_* (0.974 **).

The legacy effects of pre-drought light conditions differentially influenced rehydration outcomes in *R. pseudoacacia* (Table 4). For full-light rehydration groups (T_1_, T_3_), plants with prior full-light drought (T_1_) showed greater relative increase. For low-light rehydration groups (T_2_, T_4_), full-light drought plants (T_2_) maintained strong positive W_n_-*SLA* correlations (0.771 **), and shaded–drought plants (T_4_) showed negative associations (−0.707 *).

### 2.6. Analysis of Synergistic Effects Between Leaf Traits, Photosynthesis, and Fluorescence Parameters in P. tabuliformis and R. pseudoacacia

#### 2.6.1. Correlation of Parameters of *P. tabuliformis* in Four Treatment Groups

**Figure 7:**
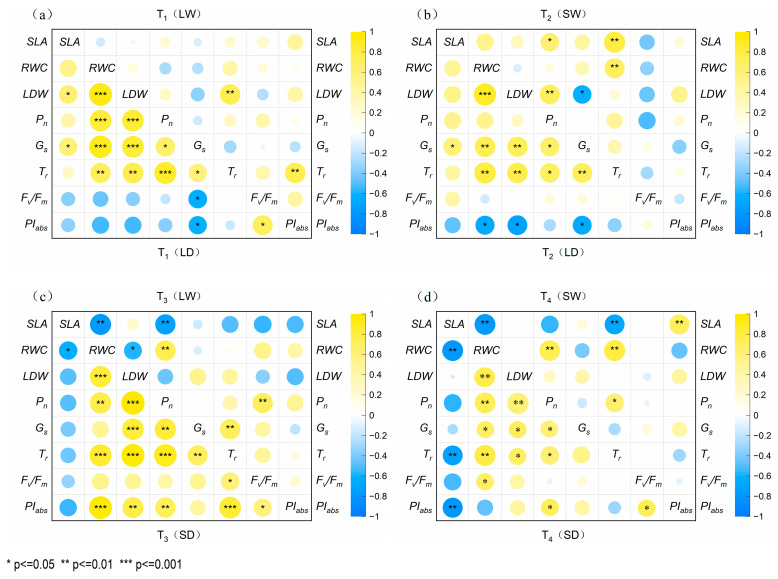
The correlation between leaf traits and photosynthetic performance of young *P. tabuliformis* under full-light and shade drought and rehydration conditions. (**a**) Correlation matrix for T_1_ (LD-LW) during the drought and rehydration phases. (**b**) Correlation matrix for T_2_ (LD-SW) during the drought and rehydration phases. (**c**) Correlation matrix for T_3_ (SD-LW) during the drought and rehydration phases. (**d**) Correlation matrix for T_4_ (SD-SW) during the drought and rehydration phases. The **lower left** part of each figure represents the drought phase of each treatment group, and the **upper right** part represents the rehydration phase. * indicates a significant correlation at the 0.05 level, ** indicates significant correlation at the 0.01 level, *** indicates significant correlation at the 0.001 level.

As shown in Figure 7a,b, parameter correlations were significantly altered by low-light rehydration following full-light drought (T_2_). In the full-light rehydration group (T_1_), *T_r_* showed highly significant positive correlations with both *LDW* and *PI_abs_* (*p <* 0.01). Low-light rehydration induced significant positive correlations between *P_n_* and *LDW*/*SLA* (*p <* 0.05), while establishing a negative correlation between *G_s_* and *LDW*.

As shown in Figure 7c,d, the pre-shaded drought stage groups exhibited distinct response patterns during rehydration. Full-light rehydration (T_3_) resulted in highly significant negative correlations (*p* < 0.01) between *SLA* and both *P_n_* and *RWC*. In contrast, low-light rehydration (T_4_) established a highly significant positive correlation (*p* < 0.01) between *SLA* and *PI_abs_*.

Under full-light rehydration (Figure 7a,c), the T_3_ group with prior shading showed inhibited *SLA* recovery, exhibiting significantly stronger negative correlations with photosynthetic parameters than the T_1_ group. Under low-light rehydration (Figure 7b,d), the T_4_ group with prior shading demonstrated significantly stronger positive *SLA*-*PI_abs_* correlations after continuous low-light rehydration than the non-shaded T_2_ group.

#### 2.6.2. Correlation of Parameters of *R. pseudoacacia* in Four Treatment Groups

**Figure 8:**
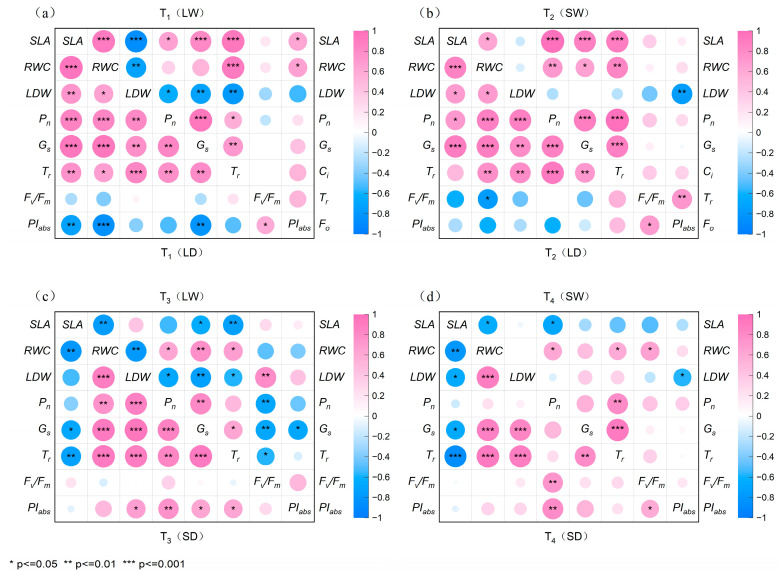
The correlation between leaf traits and photosynthetic performance of young *R. pseudoacacia* under full-light and shade drought and rehydration conditions. (**a**) Correlation matrix for T_1_ (LD-LW) during the drought and rehydration phases. (**b**) Correlation matrix for T_2_ (LD-SW) during the drought and rehydration phases. (**c**) Correlation matrix for T_3_ (SD-LW) during the drought and rehydration phases. (**d**) Correlation matrix for T_4_ (SD-SW) during the drought and rehydration phases. The lower left part of each figure represents the drought phase of each treatment group, and the upper right part represents the rehydration phase. * indicates a significant correlation at the 0.05 level, ** indicates significant correlation at the 0.01 level, *** indicates significant correlation at the 0.001 level.

As shown in Figure 8a,b, full-light rehydration (T_1_) showed significant negative correlations between *LDW* and other parameters while weakening the associations between fluorescence parameters and other physiological indicators. In contrast, low-light rehydration (T_2_) reduced the correlation strength between *LDW*/fluorescence parameters and other metrics, with *LDW* and *PI_abs_* exhibiting a highly significant negative correlation (*p <* 0.01).

As shown in Figure 8c,d, full-light rehydration (T_3_) preserved the predominant negative correlations between *SLA* and other parameters, while significantly strengthening negative relationships involving both *LDW* and *F_v_/F_m_* with other metrics. Notably, *LDW* and *F_v_/F_m_* showed a highly significant positive correlation (*p* < 0.01) under T_3_. Under low-light rehydration (T_4_), inter-parameter correlations generally weakened. However, *T_r_* and *P_n_* developed a highly significant positive relationship (*p* < 0.01), and *PI_abs_* and *LDW* showed a significant negative correlation (*p <* 0.05).

Under full-light rehydration (Figure 8a,c), the T_3_ group with prior shaded drought showed significantly stronger negative correlations between *SLA* and photosynthetic parameters than those in the T_1_ group (*p <* 0.01). Under low-light rehydration (Figure 8b,d), the continuously shaded T_4_ group had a significantly weaker negative correlation between *PI_abs_* and *LDW* than the T_2_ group.

## 3. Discussion

### 3.1. Differential Effects of Full-Light/Shade Drought and Rehydration on Leaf Traits and Photosynthetic and Fluorescence Parameters

Leaves serve as the primary sites for photosynthesis and respiration in plants, and they are the organs most sensitive to environmental changes [24]. Specific leaf area (*SLA*), defined as the ratio of leaf area to leaf dry mass, is a key indicator reflecting a plant’s capacity to utilize light resources. Under shading, plants often increase their *SLA* to enhance light capture, thereby improving the efficiency of light—energy capture per unit of leaf dry weight [25]. Previous research has demonstrated significant differences in shading—adaptation strategies between evergreen and deciduous species. Deciduous saplings are more sensitive than evergreen ones to changes in light effectiveness in growth characteristics, *SLA*, and dry-weight allocation [26]. In the present study, during the drought phase, shading assisted *P. tabuliformis* in maintaining its *SLA* but led to a substantial increase in the *SLA* of *R. pseudoacacia*. During the post-drought rehydration period, appropriate shading may be beneficial for *R. pseudoacacia*. It can mitigate photoinhibition at the initial stage of rehydration and promote leaf-area expansion, thus enhancing the plant’s adaptability to fluctuating-water environments.

Leaf relative water content (*RWC*) represents a critical indicator of plant water status and drought tolerance. As stress intensifies, *RWC* exhibits a downward trend. In the current investigation, the leaf *RWC* of *P. tabuliformis* and *R. pseudoacacia* demonstrated a significant decrease with the escalation of drought stress, which aligns with findings from prior studies [27,28]. During the drought phase, shading exacerbated the suppression of *RWC* in *R. pseudoacacia*, whereas it exerted a lesser impact on *P. tabuliformis*. The results of the rehydration experiment indicated that a stable shaded environment facilitated the recovery of *RWC* in *P. tabuliformis*, potentially attributed to the lag in chloroplast elastic regulation in this species.

In the present study, drought stress significantly reduced the leaf dry weight (*LDW*) of *P. tabuliformis* and *R. pseudoacacia*, with additional shading exacerbating this reduction. This finding is consistent with previous investigations [29,30]. Notably, the *LDW* of *P. tabuliformis* demonstrated high sensitivity to shading stress under drought conditions, whereas *R. pseudoacacia* exhibited minimal *LDW* variation. This suggests that for *P. tabuliformis* in drought-prone habitats, continuous light availability is critical to maintain the carbon source required for *LDW* accumulation. In contrast, *R. pseudoacacia* appears to accumulate leaf dry weight by regulating leaf growth, increasing specific leaf area, and optimizing light capture in shaded environments. Following rewatering, the *LDW* of both species failed to recover to control levels, which may be attributed to irreversible adjustments in carbon allocation strategies [31,32,33].

Drought and shading are key environmental factors that suppress plant photosynthesis. Drought stress induces stomatal closure, reduces photosynthetic enzyme activity, and decreases CO_2_ permeability of the cuticular cell wall, collectively leading to suppressed photosynthetic capacity [34]. Excessively low-light intensities also hinder the accumulation of photosynthetic products [35]. In this study, as soil water content declined, the photosynthetic parameters—net photosynthetic rate (*P_n_*), stomatal conductance (*G_s_*), and transpiration rate (*T_r_*)—of young trees of all species gradually decreased, aligning with previous research results [36,37]. When shading occurs during drought, it exacerbates drought damage and stomatal closure in *P. pseudotabulacea*. In contrast, *R. pseudoacacia* uses shading to mitigate photo-oxidative stress. Compared to *P. tabuliformis*, *P. pseudotabulacea* exhibits more extreme stomatal restriction to avoid photodamage risks.

Changes in transpiration rates indicate that stomatal regulation in *P. tabuliformis* and *R. pseudoacacia* is more sensitive to light-environment changes. This reflects a conservative strategy in both species to maintain water transport through a shaded environment during drought stress [38,39]. The *P_n_* of both tree species did not recover to the control level after rewatering. The *G_s_* recovery of *P. tabuliformis* in the shaded drought stage groups (T_3_, T_4_) was significantly lower than that in the full-light drought groups (T_1_, T_2_), a discrepancy attributed to the delayed recovery of chloroplast ultrastructure damage and Rubisco enzyme activity [40,41]. However, *R. pseudoacacia* showed a super compensatory effect after rewatering in the full-light drought group [40,42]. In *P. pseudotabulacea* and *R. pseudoacacia*, resetting the light environment after rewatering may positively regulate *P. pseudotabulacea*’s stomatal function recovery. Transpiration recovery results show that a stable shaded environment favors water transport system restoration. The stomatal regulation strategy and transpiration recovery of *R. pseudoacacia* suggest the presence of an environmental memory effect [11,43].

In plant physiological ecology, *F_v_/F_m_* serves as a key indicator of PSII primary light–energy conversion efficiency in leaves, which is typically in the range of 0.75 to 0.85 for normally growing plants [44]. In this study, the *F_v_/F_m_* of both *P. tabuliformis* and *R. pseudoacacia* remained above 0.75, indicating strong adaptability under the given conditions. Despite fluctuations, *F_v_/F_m_* showed overall stability, suggesting that plant growth could normalize if stressors are removed [33,45]. *PI_abs_* indicates the efficiency of light–energy utilization during photosynthesis and the ability to adapt to environmental stresses. In this study, under full-light drought, *P. tabuliformis* and *R. pseudoacacia* showed significantly more effectively *PI_abs_* than under shade drought. Additionally, the pre-drought environmental conditions influenced the recovery dynamics during rehydration. This implies healthier photosynthetic apparatus and better photosynthetic acclimatization under full-light drought, while shading worsened drought damage [46,47].

### 3.2. Correlation of Full-Light/Shade Drought and Rehydration Time Dynamics with Physiological Response

In the drought stage, the degree of photosynthetic inhibition, water loss, and photosystem damage in plants progressively intensifies with the prolongation of stress duration. The efficiency of recovery after rehydration is contingent upon the physiological repair capacity within different time windows [48,49,50]. In this study, drought duration (D_n_) exhibited a significant negative correlation with most physiological indices in both *P. tabuliformis* and *R. pseudoacacia* (*p* < 0.01). This indicates that the cumulative effect of stress intensifies with increasing drought duration, which is consistent with the findings of Diawara [51], Nawaz [52], and Li [53]. *P. tabuliformis* showed no significant positive correlation between *SLA* and D_n_ under shading drought. This suggests that the conservative light–energy utilization strategy of *P. tabuliformis* relies more on functional stability than morphological adjustments—a finding also supported by Wang [54] and Deng [55]. In contrast, *R. pseudoacacia* exhibited a highly significant positive correlation between *SLA* and D_n_ under shading drought stages. This implies that *R. pseudoacacia* enhances light-capture capacity through morphological plasticity to partially offset photosystem damage caused by drought stress [56].

During the rehydration phase, the light environment regulated recovery efficiency in a species-specific way. For *P. tabuliformis* during short-term rehydration (W_0.25–0.5_), full-light rehydration significantly promoted the rapid recovery of stomatal conductance (*G_s_*), while continuous low-light rehydration (W_1–7_) inhibited the restoration of its photosystem. This suggests that the photosystem restoration of *P. tabuliformis* is highly dependent on sustained light input during the later stages of rehydration [57]. Conversely, *R. pseudoacacia* showed higher light–energy utilization efficiency under shaded and rehydration conditions, which might be related to shading alleviating photo-oxidative stress and maintaining PSII stability [58].

In addition, the legacy effects of pre-existing light environments significantly impacted the rewetting recovery potential. Following rehydration of the pre-shaded drought group, the negative correlation between *SLA* and photosynthetic parameters was stronger in *P. tabuliformis* (T_3_) than in the full-light drought group (T_1_). This indicates that pre-stress shading weakened the synergistic restoration of leaf morphology and photosynthetic function. In *R. pseudoacacia*, shading and rewetting following the initial full-light drought (T_2_) enhanced the positive correlation between *SLA* and *P_n_*, thereby optimizing resource allocation efficiency. This confirms the flexibility of its spatio-temporal compensation strategy.

### 3.3. Synergistic Mechanisms of Leaf Traits and Photosynthetic Performance Under Full-Light/Shade Drought and Recurring Water

The ability of plants to survive under multiple stresses is intimately associated with the synergistic efficiency of leaf traits and photosynthetic performance [59,60]. This study reveals that *P. tabuliformis* and *R. pseudoacacia* employ very different strategies to mitigate combined drought and shade stress. *P. tabuliformis* prioritizes protecting photosystem integrity by maintaining stable leaf morphology, while *R. pseudoacacia* achieves efficient resource use through rapid adjustments of morphological plasticity and physiological flexibility [54]. This divergence is deeply rooted in the evolutionary trajectories of the two species. *P. tabuliformis*, an evergreen conifer with a needle-like structure, has limited plasticity adjustments and relies on functional stability. Its strategy focuses on maintaining leaf water content to adapt to drought and sustain normal physiological activity [61]. In contrast, *R. pseudoacacia*, a deciduous broad-leaved tree, can expand leaf area to enhance light–energy capture and balance light and water use through dynamic stomatal regulation [62,63]. However, shading only slowed the decline in photosynthesis of *R. pseudoacacia* under drought, still causing irreversible damage and impaired its post-drought photosynthetic recovery under normal moisture conditions. These findings validate Holmgren’s “trade-off theory” [14] and reveal species-specific ‘temporal-spatial compensatory strategies’ in stress synergism.

*P. tabuliformis* exhibits more dry matter by increasing photosynthesis under shading and rehydration, potentially mediated by leaf area expansion to facilitate light capture [8,64]. However, large stomatal openings may increase water loss, thereby imposing trade-offs on dry matter accumulation [65]. Under the combined shade–rewatering treatment, the *P_n_* of *R. pseudoacacia* was markedly suppressed. This “down-regulation” alleviated photoinhibition by reducing excessive excitation pressure on PSII reaction centers [66]. The shade-induced increase in *SLA* enhances light interception, yet this increment constrains the supply of ATP for stomatal regulation in low-light environments. Consequently, psbA transcription–translation slows, hindering D1 protein cleavage and replacement, and prolonging the restoration of the PSII repair cycle [67,68]. As a result, although leaves capture more photons and the proportion of fully functional PSII declines, photochemical quenching (ΦPSII) and Pn remain low, and the net accumulation of photosynthetic products actually decreases.

*P. tabuliformis* enhanced its low-light adaptive response through morphological and physiological adjustments following shading exposure, increasing the efficiency of photosynthesis and growth potential under rehydrated conditions [69]. At this stage, the guard cells of *P. tabuliformis* moderately restrict stomatal aperture, thereby balancing carbon gain with water loss [70]. However, shading pre-stress exacerbated the asynchronous nature of *R. pseudoacacia* leaf anatomy reconstruction and photosynthetic function recovery. This may be because shading enhances its light-absorbing capacity, yet insufficient light limits the ATP supply to Rubisco activase, lowering photosynthetic enzyme activity and reducing the efficiency of converting light into dry-mass gain; this reduction is partially alleviated after rewatering [71,72,73].

## 4. Materials and Methods

### 4.1. Materials

The study used three-year-old saplings of two typical Beijing urban trees: *P. tabuliformis* and *R. pseudoacacia*. In late March 2021, the saplings were transplanted into 45 cm × 45 cm pots with soil from Beijing’s Xishan Mountain. Placed in the nursery of the Institute of Forestry and Fruit Tree Research, Beijing Academy of Agricultural and Forestry Sciences (39°59′ N, 116°13′ E, 88 m above sea level), the saplings were grown under uniform light and water conditions.

### 4.2. Treatment Design

Saplings Preparation: At the end of February 2021, a total of 130 three-year-old saplings of *P. tabuliformis* and *R. pseudoacacia* were selected and planted in 45 cm × 45 cm plastic pots measuring at Institute of Forestry and Fruit Research, Beijing Academy of Agriculture and Forestry Sciences. During the acclimatization period, the saplings were irrigated to the 80–90% field capacity of potted soil to homogenize the height and basal diameter of saplings of two tree species, ensuring the uniformity of growth and physiological consistency. During the entire domestication and treatment period, the air temperature and relative humidity were continuously recorded every 1 h using the Weather Meter meteorological Station. The average daily temperature was 25.4 °C, and the average relative humidity was 50.3%. The environmental conditions for the growth of plant seedlings are natural light.

Treatment Design: In July 2021, all the above potted saplings were moved to a plastic shade house with both rain and shade functions. Four treatment groups and one control group (CK) were set up to carry out the experiment, with 30 young trees in each group and 10 in the control group. The treatment groups were T_1_ (LD-LW): 100% natural light radiation (full-light) throughout, with soil dried to wilting point (drought) followed by rewatering to field capacity (*FC*) of potted soil (recovery); T_2_ (LD-SW): full-light during drought, switched to low-light during recovery; T_3_ (SD-LW): 20% natural light radiation (low-light) during drought, switched to full-light during recovery; and T_4_ (SD-SW): low-light throughout, with soil rewatered to field capacity post-wilting.

The above treatments all included two stages: drought and rewatering. In the drought stage, 30 tree saplings of *P. tabuliformis* and *R. pseudoacacia* were respectively placed in a full-light or low-light shade house, and after irrigating to 80–90% field capacity of potted soil, watering was withheld to allow natural drought stress until the soil volumetric water content of the potted soil reached the preset threshold. During the rewatering stage, drought-stressed saplings were irrigated to field capacity under either full- or low-light, with soil moisture maintained at 80–90% field capacity thereafter. Shade condition was achieved using HDPE material shading nets, which have a shading rate of 80% and do not change the spectral characteristics, with the light intensity monitored by a TES1339 illuminance meter during shading. Drought condition was induced via natural soil drying, and the soil volumetric water content (*SWC*) of potted soil was measured using a portable soil sensor (WET-2, Delta T, Richfield, WI, USA).

Sampling Nodes: For the four treatment groups, the drought stage had three sampling points: pre-drought (D_1_, *SWC* at about 20–26%), mid-drought (D_2_, *SWC* at about 12–14%), and late drought (D_3_, *SWC* at about 4–6%). After the drought stage, rewatering was performed, and the post-drought rewatering stage had four sampling time points: 6 h (W_1_), 12 h (W_2_), 24 h (W_3_), and 7 days (W_4_) after rewatering. During the rewatering stage, *SWC* was monitored in real-time to maintain soil at 80–90% *FC*. Sampling and measurements were taken at each sampling nodes (D_1_, D_2_, D_3_, W_1_, W_2_, W_3_, W_4_) to analyze the growth and photosynthetic responses of saplings to different treatments. The *SWC* values for each sampling node were consistent across the four treatment groups.

### 4.3. Indicator Measurement

Specific Leaf Area (*SLA*): At each sampling node during the drought and rehydration stages, 20 fully expanded leaves were collected from the upper-middle and sunny-side canopy of each potted sapling of both species. These leaves were scanned indoors, and leaf area was measured using IMAGEJ 1.53 software (Wayne Rasband, National Institute of Health, Bethesda, MD, USA).

Leaf dry weight (*LDW*) and leaf relative water content (*RWC*): At each sampling time point for each tree species, leaves to be tested were randomly selected and quickly collected and sealed to prevent water evaporation. A portion of each leaf (avoiding the midrib) was cut and weighed to determine fresh weight (*LFW*). The sample was then killed in a 105 °C oven for 30 min. Subsequently, the temperature was lowered to 80 °C, and the sample was dried in an oven for 48 h until a constant weight was achieved. After cooling, the weight was measured to obtain the *LDW*.

$$RWC=\frac{LFW-LDW}{LFW}\times 100\%$$

Leaf photosynthetic parameters: During the experiments under shaded, full-light, and control conditions, the photosynthetic parameters of young trees [net photosynthetic rate (*P_n_*, µmol·m^−2^·s^−1^), transpiration rate (*T_r_*, mmol*·*m^−2^·s^−1^), and stomatal conductance (*G_s_*, mol·m^−2^·s^−1^)] were measured between 8:00 and 10:00 a.m. on sunny days at each sampling time point using a CI-340 portable photosynthesis meter, CID Bio-Science Inc., Camas, WA, USA (the leaf age is selected as fully developed mature leaves of the current year).

Chlorophyll fluorescence parameters: At each sampling time point, chlorophyll fluorescence parameters of young trees were measured between 8:00 and 10:00 a.m. using a high-speed continuous-excitation fluorometer (Handy PEA, Norfolk, UK). Parameters included the maximum photochemical efficiency (*F_v_/F_m_*) and photosynthetic performance index (*PI_abs_*). These measurements helped evaluate the distinct effects of combined shade and drought stresses on the photosynthetic apparatus and the restoration process during rehydration.

### 4.4. Data Processing

All raw data were organized in Excel 2021 and subsequently analyzed in SPSS 24.0. Differences within and among groups in Section 2.1, Section 2.2 and Section 2.3 were tested for significance using the LSD method. Section 2.4 employed a two-factor (drought/rewatering × light) linear mixed-effects model to examine the main effects and interactions; indicators exhibiting significant interactions were further subjected to simple-effects analysis with Bonferroni correction. Correlation analyses in Section 2.5 and Section 2.6 were conducted using Pearson’s method. All figures were generated with Origin 2024.

## 5. Conclusions

This study elucidates the differential mechanisms through which combined drought and shading stress impact leaf traits and photosynthetic performance in juvenile *P. tabuliformis* and *R. pseudoacacia*. Combined drought and shade reduced *SLA*, *RWC,* and *LDW* in both juvenile *P. tabuliformis* and *R. pseudoacacia*, but shade buffered these declines only in *R. pseudoacacia* by relieving photoinhibition, demonstrating its greater morphological plasticity. Shade exacerbated drought-driven reductions in *P_n_* and *G_s_* in *P. tabuliformis* while mitigating them in *R. pseudoacacia*, underscoring divergent light-use strategies. Upon rehydration, plants previously subjected to shaded drought recovered less than those under full-light drought, especially *P. tabuliformis*, indicating that shade hampers PSII repair and prolongs drought damage. These findings suggest deploying shade-tolerant *R. pseudoacacia* in low-light urban microhabitats and reserving sun-dependent *P. tabuliformis* for open areas, thereby optimizing light–water use efficiency, enhancing drought resilience and improving the ecological restoration potential of Beijing’s urban forests. In the future, in-depth research is needed on the synergistic changes of biochemical indicators such as plant osmolytes, antioxidant enzymes, and gene expression to unravel the underlying mechanisms.

## Figures and Tables

**Table 1:** Specific leaf area (*SLA*, cm^2^/g) of *P. tabuliformis* and *R. pseudoacacia* leaves under four treatment groups during continuous drought and subsequent rewatering.

Species	Stage	Specific Leaf Area (cm^2^/g)			
		T_1_ (LD-LW)	T_2_ (LD-SW)	T_3_ (SD-LW)	T_4_ (SD-SW)
*P. tabuliformis*	CK	54 ± 5.51 a	54 ± 5.51 a	53 ± 5.29 d	53 ± 5.29 bc
	D_1_	54 ± 5.13 a	54 ± 5.86 a	55 ± 5.03 cd	51 ± 3.21 c
	D_2_	53 ± 2.00 Aa	48 ± 2.00 Cabcd	57 ± 2.00 Abcd	52 ± 2.00 Babc
	D_3_	38 ± 4.93 Cb	45 ± 4.04 Bbc	59 ± 1.53 Abc	59 ± 2.08 Aa
	W_1_	37 ± 10.21 Cb	41 ± 2.08 Cc	74 ± 3.79 Aa	57 ± 1.53 Bab
	W_2_	37 ± 2.65 Db	45 ± 3.06 Cbc	62 ± 2.08 Ab	55 ± 1.00 Babc
	W_3_	42 ± 2.52 Bb	52 ± 2.65 Aab	52 ± 2.00 Ade	53 ± 1.53 Abc
	W_4_	48 ± 5.69 b	52 ± 4.58 ab	47 ± 1.00 e	51 ± 1.53 c
*R. pseudoacacia*	CK	155 ± 4.93 b	155 ± 4.93 de	159 ± 4.04 d	159 ± 4.04 d
	D_1_	157 ± 1.53 Cb	165 ± 6.03 Ccd	187 ± 6.81 Ac	205 ± 6.66 Bc
	D_2_	134 ± 3.61 Cc	137 ± 4.36 Cf	194 ± 4.16 Ab	224 ± 5.03 Bc
	D_3_	111 ± 1.00 Ce	118 ± 4.16 Cg	214 ± 1.53 Aa	245 ± 8.14 Ba
	W_1_	125 ± 5.00 Cd	152 ± 10.69 Bd	222 ± 7.55 Ab	229 ± 9.02 Aa
	W_2_	158 ± 4.73 Cb	171 ± 3.51 Bc	214 ± 4.58 Ab	223 ± 7.02 Aa
	W_3_	157 ± 2.52 Cb	192 ± 7.21 Bb	215 ± 5.00 Ab	221 ± 7.81 Aa
	W_4_	169 ± 2.52 Ba	200 ± 10.58 Aa	204 ± 6.66 Ac	206 ± 3.21 Ab

The data is the mean ± standard deviation. D_x_ represents the drought stage, and W_x_ represents the rehydration stage. Different uppercase letters in the figure indicate significant differences between the T_1_, T_2_, T_3_, and T_4_ groups at each sampling node at the *p* < 0.05 level, while different lowercase letters indicate significant differences within the same group at different nodes at the *p* < 0.05 level.

**Table 2:** The interaction effect (*p* value) of shading during the drought period and shading during the rehydration period on various physiological indicators.

Species	Parameter	Drought × Light	Rewatering × Light	Species	Parameter	Drought × Light	Rewatering × Light
*P. tabuliformis*	*SLA*	**0.000**	0.550	*R. pseudoacacia*	*SLA*	0.581	0.922
	*RWC*	0.344	0.150		*RWC*	0.593	0.575
	*LDW*	**0.025**	**0.007**		*LDW*	**0.000**	**0.001**
	*P_n_*	0.235	**0.015**		*P_n_*	0.452	0.235
	*G_s_*	**0.000**	0.868		*G_s_*	0.222	0.844
	*T_r_*	0.887	0.675		*T_r_*	0.125	0.855
	*F_v_/F_m_*	**0.000**	0.518		*F_v_/F_m_*	**0.029**	**0.006**
	*PI_abs_*	**0.000**	**0.029**		*PI_abs_*	0.295	0.549

The data is the *p*-value of the interaction term output by the linear mixed-effects model; “drought × light” indicates “the interaction between drought and light”, and “rewatering × light” indicates “the interaction between rewatering and light”; The significance level α = 0.05; The bold value *p* < 0.05 indicates a significant interaction.

**Table 3:** Correlation between drought-restoration duration and physiological indices of *P. tabuliformis*.

Pearson		*P. tabuliformis*							
		*SLA*	*RWC*	*LDW*	*P_n_*	*G_s_*	*T_r_*	*F_v_/F_m_*	*PI_abs_*
T_1_	D_n_	−0.586 *	**−0.941** **	**−0.943** **	**−0.929** **	**−0.852** **	**−0.875** **	0.332	0.476
	D_1–2_	0.464	**−0.956** **	−0.762	−0.751	−0.913 *	−0.878 *	0.768	0.778
	D_2–3_	**−0.954** **	−0.898 *	−0.805	−0.722	−0.797	−0.266	0.373	0.646
	Wn	**0.782** **	−0.084	−0.163	0.241	0.058	0.341	0.545	0.511
	W_0.25–0.5_	−0.027	0.226	**0.956** **	0.545	−0.560	**0.959** **	0.497	0.900 *
	W_1–7_	0.845 *	−0.468	−0.787	0.490	−0.224	0.243	**0.953** **	0.707
T_2_	D_n_	−0.672 *	**−0.888** **	**−0.862** **	−0.707 *	**−0.873** **	**−0.859** **	−0.146	0.703 *
	D_1–2_	0.036	−0.796	−0.746	−0.818 *	−0.536	**−0.996** **	−0.197	−0.708
	D_2–3_	−0.409	−0.713	−0.984 **	0.867 *	−0.355	**−0.962** **	0.000	0.859 *
	W_n_	0.428	0.480	−0.187	0.054	0.553	0.658 *	0.140	0.312
	W_0.25–0.5_	0.601	0.802	−0.322	0.105	0.394	0.915 *	−0.260	−0.206
	W_1–7_	0.000	0.433	−0.852 *	**−0.918** **	0.650	0.728	0.459	0.664
T_3_	D_n_	0.608 *	**−0.899** **	**−0.942** **	**−0.917** **	−0.694 *	**−0.951** **	−0.578 *	**−0.884** **
	D_1–2_	0.258	−0.850 *	**−0.931** **	**−0.959** **	0.758	−0.784	0.535	−0.916 *
	D_2–3_	0.676	−0.590	−0.824 *	−0.328	−0.755	−0.784	−0.899 *	−0.614
	W_n_	**−0.712** **	0.593 *	−0.291	0.592 *	0.157	0.397	0.378	0.707 *
	W_0.25–0.5_	**−0.923** **	−0.074	0.836 *	0.060	**0.955** **	0.891 *	0.620	−0.212
	W_1–7_	−0.889 *	0.104	0.086	0.364	0.827 *	0.868 *	0.234	0.735
T_4_	D_n_	0.532	**−0.907** **	**−0.913** **	**−0.906** **	**−0.860** **	**−0.841** **	−0.595 *	−0.647 *
	D_1–2_	0.151	−0.848 *	−0.903 *	−0.751	0.443	−0.626	0.106	−0.619
	D_2–3_	0.917 *	−0.804	−0.348	−0.884 *	**−0.958** **	**−0.955** **	**−0.974** **	**−0.974** **
	W_n_	**−0.720** **	0.541	−0.033	0.096	−0.455	0.454	0.013	**−0.871** **
	W_0.25–0.5_	−0.742	0.795	−0.872 *	0.814 *	−0.640	0.579	0.221	−0.708
	W_1–7_	−0.626	0.357	−0.825 *	**−0.923** **	−0.588	0.683	0.530	**−0.959** **

The bold value *p* < 0.05 indicates a significant interaction. * indicates a significant correlation at the 0.05 level (two-tailed), ** indicates significant correlation at the 0.01 level (two-tailed).

**Table 4:** Correlation between drought-restoration duration and physiological indices of *R. pseudoacacia*.

Pearson		*R. pseudoacacia*							
		*SLA*	*RWC*	*LDW*	*P_n_*	*G_s_*	*T_r_*	*F_v_/F_m_*	*PI_abs_*
T_1_	D_n_	**−0.922** **	**−0.889** **	**−0.872** **	**−0.920** **	**−0.944** **	**−0.908** **	0.063	0.673 *
	D_1–2_	**−0.982** **	**−0.959** **	−0.479	**−0.946** **	−0.872 *	0.099	0.745	0.814 *
	D_2–3_	**−0.983** **	−0.850 *	−0.573	−0.014	**−0.958** **	−0.993 **	0.114	0.606
	W_n_	0.633 *	0.236	−0.650 *	**0.818** **	**0.822** **	0.373	0.202	0.339
	W_0.25–0.5_	**0.973** **	0.974 **	−0.836 *	−0.673	0.887 *	**0.995** **	0.666	0.799
	W_1–7_	**0.946** **	−0.071	−0.736	0.911 *	0.853 *	−0.668	0.868 *	0.478
T_2_	D_n_	**−0.847** **	**−0.944** **	**−0.819** **	**−0.941** **	**−0.922** **	**−0.870** **	−0.686 *	−0.533
	D_1–2_	**−0.958** **	−0.803	−0.864 *	**−0.923** **	**−0.984** **	−0.806	0.372	0.489
	D_2–3_	**−0.937** **	−0.835 *	0.307	−0.726	**−0.961** **	**−0.966** **	−0.808	−0.777
	W_n_	**0.771** **	0.255	−0.346	**0.740** **	0.672 *	0.590 *	0.497	0.249
	W_0.25–0.5_	0.830 *	0.879 *	−0.637	**0.954** **	**0.975** **	**0.987** **	0.788	**0.934** **
	W_1–7_	0.755	−0.385	−0.885 *	0.845 *	0.336	0.757	**0.956** **	0.870 *
T_3_	D_n_	**0.963** **	**−0.892** **	−0.675 *	−0.531	**−0.806** **	**−0.853** **	0.216	−0.285
	D_1–2_	0.892 *	−0.906 *	−0.867 *	−0.493	−0.697	**−0.973** **	0.840 *	−0.491
	D_2–3_	0.885 *	−0.868 *	**−0.951** **	−0.889 *	**−0.952** **	**−0.982** **	−0.784	−0.904 *
	W_n_	**−0.830** **	**0.731** **	−0.501	0.577 *	0.464	**0.783** **	−0.376	0.005
	W_0.25–0.5_	−0.414	**0.940** **	−0.392	−0.218	0.436	−0.480	0.742	−0.347
	W_1–7_	−0.872 *	0.873 *	0.426	0.196	−0.407	0.783	0.784	0.597
T_4_	D_n_	**0.958** **	**−0.902** **	**−0.793** **	−0.043	**−0.714** **	**−0.927** **	0.067	−0.053
	D_1–2_	0.605	−0.656	**−0.946** **	**0.948** **	0.499	−0.826 *	0.515	0.833 *
	D_2–3_	**0.968** **	**−0.967** **	**−0.969** **	**−0.973** **	**−0.982** **	**−0.929** **	−0.739	−0.901 *
	W_n_	−0.707 *	0.675 *	−0.247	**0.966** **	0.519	**0.744** **	0.600 *	0.384
	W_0.25–0.5_	−0.617	**0.935** **	0.180	−0.205	−0.720	−0.335	0.831 *	0.066
	W_1–7_	−0.743	0.813 *	−0.729	**0.974** **	−0.468	0.758	0.814 *	0.485

The bold value *p* < 0.05 indicates a significant interaction. * indicates a significant correlation at the 0.05 level (two-tailed), ** indicates significant correlation at the 0.01 level (two-tailed).

## Data Availability

The raw data supporting the conclusions of this article will be made available by the authors on request.

## Supplementary Materials

The following supporting information can be downloaded at: https://www.mdpi.com/article/10.3390/plants14182825/s1, Table S1: Simple-effects analysis of *SLA*, *G_s_*, *F_v_/F_m_* and *PI_abs_* in *P. tabuliformis* during drought under full/low light conditions; Table S2: Simple-effects analysis of *LDW*, *P_n_* and *PI_abs_* recovery in *P. tabuliformis* during re-watering under full/low light conditions; Table S3: Simple-effects analysis of *LDW* and *F_v_/F_m_* in *R. pseudoacacia* during drought under full/low light conditions; Table S4: Simple-effects analysis of *LDW* and *F_v_/F_m_* recovery in *R. pseudoacacia* during rewatering under full/low-light conditions.
