# Supplementary material for: Synergistic Interactions Between Leaf Traits and Photosynthetic Performance in Young Pinus tabuliformis and Robinia pseudoacacia Trees Under Drought and Shade
Source: Plants (Basel). 2025 Sep 10;14(18):2825. doi: 10.3390/plants14182825 (PMC12473763; doi:10.3390/plants14182825)
Supplement: Supplementary file 1 [file plants-14-02825-s001.zip › plants-3804412-supplementary.pdf]

Table S1 Simple-effects analysis of *SLA*, G_s, F_v/F_m and PI_{abs} in *P. tabuliformis* during drought under full/low light conditions

Drought					
	Full light			Low light	
Parameter	Treatment Stage	Significance	Parameter	Treatment Stage	Significance
	$D_1 \times D_2$	1.000		$D_1 \times D_2$	1.000
SLA	$D_2 \times D_3$	**0.001**	*SLA*	$D_2 \times D_3$	0.170
	$D_3 \times D_1$	**0.000**		$D_3 \times D_1$	0.064
	$D_1 \times D_2$	**0.000**		$D_1 \times D_2$	0.223
G_s	$D_2 \times D_3$	0.120	G_s	$D_2 \times D_3$	**0.004**
	$D_3 \times D_1$	0.000		$D_3 \times D_1$	0.316
	$D_1 \times D_2$	0.269		$D_1 \times D_2$	1.000
F_v/F_m	$D_2 \times D_3$	1.000	F_v/F_m	$D_2 \times D_3$	**0.001**
	$D_3 \times D_1$	**0.046**		$D_3 \times D_1$	**0.006**
	$D_1 \times D_2$	0.052		$D_1 \times D_2$	**0.022**
PI_{abs}	$D_2 \times D_3$	0.198	PI_{abs}	$D_2 \times D_3$	**0.001**
	$D_3 \times D_1$	**0.000**		$D_3 \times D_1$	**0.000**

Values are Bonferroni-adjusted *p*-values for pairwise contrasts among drought stages D_1, D_2 and D_3 (n = 10). Bold $p < 0.05$ indicates that the two drought stages differ significantly within the same light regime.

Table S2 Simple-effects analysis of *LDW*, P_n and PI_{abs} recovery in *P. tabuliformis* during re-watering under full/low light conditions

Rewatering					
	Full light			Low light	
Parameter	Treatment Stage	Significance	Parameter	Treatment Stage	Significance
	$W_1 \times W_2$	0.072		$W_1 \times W_2$	0.199
	$W_1 \times W_3$	0.433		$W_1 \times W_3$	1.000
	$W_1 \times W_4$	1.000		$W_1 \times W_4$	1.000
LDW	$W_2 \times W_3$	1.000	*LDW*	$W_2 \times W_3$	**0.010**
	$W_2 \times W_4$	0.410		$W_2 \times W_4$	0.708
	$W_3 \times W_4$	1.000		$W_3 \times W_4$	0.503
	$W_1 \times W_2$	1.000		$W_1 \times W_2$	0.877
	$W_1 \times W_3$	1.000		$W_1 \times W_3$	**0.000**
	$W_1 \times W_4$	0.450		$W_1 \times W_4$	0.307
P_n	$W_2 \times W_3$	1.000	P_n	$W_2 \times W_3$	**0.008**
	$W_2 \times W_4$	1.000		$W_2 \times W_4$	1.000
	$W_3 \times W_4$	1.000		$W_3 \times W_4$	**0.036**
	$W_1 \times W_2$	1.000		$W_1 \times W_2$	0.448
	$W_1 \times W_3$	1.000		$W_1 \times W_3$	1.000
	$W_1 \times W_4$	0.119		$W_1 \times W_4$	0.512
PI_{abs}	$W_2 \times W_3$	1.000	PI_{abs}	$W_2 \times W_3$	0.724
	$W_2 \times W_4$	1.000		$W_2 \times W_4$	1.000
	$W_3 \times W_4$	1.000		$W_3 \times W_4$	0.819

Values are Bonferroni-adjusted *p*-values for pairwise contrasts among drought stages W_1, W_2, W_3 and W_4 (n = 10). Bold $p < 0.05$ indicates that the two rewatering stages differ significantly within the same light regime.

Table S3 Simple-effects analysis of *LDW* and F_v/F_m in *R. pseudoacacia* during drought under full/low light conditions

Drought					
	Full light			Low light	
Parameter	Treatment Stage	Parameter	Treatment Stage	Parameter	Treatment Stage
	$D_1 \times D_2$	**0.009**		$D_1 \times D_2$	**0.000**
LDW	$D_2 \times D_3$	**0.000**	*LDW*	$D_2 \times D_3$	**0.000**
	$D_3 \times D_1$	**0.003**		$D_3 \times D_1$	**0.000**
	$D_1 \times D_2$	0.319		$D_1 \times D_2$	0.063
F_v/F_m	$D_2 \times D_3$	0.193	F_v/F_m	$D_2 \times D_3$	**0.019**
	$D_3 \times D_1$	1.000		$D_3 \times D_1$	1.000

Values are Bonferroni-adjusted *p*-values for pairwise contrasts among drought stages D_1, D_2 and D_3 (n = 10). Bold $p < 0.05$ indicates that the two drought stages differ significantly within the same light regime.

Table S4 Simple-effects analysis of *LDW* and F_v/F_m recovery in *R. pseudoacacia* during rewatering under full/low-light conditions

Rewatering					
	Full light			Low light	
Parameter	Treatment Stage	Parameter	Treatment Stage	Parameter	Treatment Stage
	$W_1 \times W_2$	0.560		$W_1 \times W_2$	1.000
	$W_1 \times W_3$	**0.009**		$W_1 \times W_3$	0.245
	$W_1 \times W_4$	**0.003**		$W_1 \times W_4$	1.000
LDW	$W_2 \times W_3$	0.601	*LDW*	$W_2 \times W_3$	**0.014**
	$W_2 \times W_4$	0.241		$W_2 \times W_4$	1.000
	$W_3 \times W_4$	1.000		$W_3 \times W_4$	**0.012**
	$W_1 \times W_2$	0.247		$W_1 \times W_2$	0.002
	$W_1 \times W_3$	**0.013**		$W_1 \times W_3$	1.000
	$W_1 \times W_4$	1.000		$W_1 \times W_4$	**0.000**
F_v/F_m	$W_2 \times W_3$	**0.000**	F_v/F_m	$W_2 \times W_3$	**0.001**
	$W_2 \times W_4$	**0.045**		$W_2 \times W_4$	1.000
	$W_3 \times W_4$	0.082		$W_3 \times W_4$	**0.000**

Values are Bonferroni-adjusted *p*-values for pairwise contrasts among drought stages W_1, W_2, W_3 and W_4 (n = 10). Bold $p < 0.05$ indicates that the two rewatering stages differ significantly within the same light regime.
